# Pharmaceutical and Therapeutic Applications of Royal Jelly for Ocular Surface Diseases: A Comprehensive Review

**DOI:** 10.18502/jovr.v20.16705

**Published:** 2025-10-30

**Authors:** Mojtaba Mortazavi, Mahmood Nejabat, Mohammad Hashem Hashempur, Roghayyeh Baghban

**Affiliations:** ^1^Department of Biotechnology, Institute of Science and High Technology and Environmental Sciences, Graduate University of Advanced Technology, Kerman, Iran; ^2^Poostchi Ophthalmology Research Center, Department of Ophthalmology, School of Medicine, Shiraz University of Medical Sciences, Shiraz, Iran; ^3^Research Center for Traditional Medicine and History of Medicine, Department of Persian Medicine, School of Medicine, Shiraz University of Medical Sciences, Shiraz, Iran

**Keywords:** Integrative Medicine, Ocular Surface Diseases, Royal Jelly, Traditional Persian Medicine, Treatment

## Abstract

Ocular surface diseases (OSDs) are conditions that affect the eye's surface layers, including the cornea, conjunctiva, and glandular network, causing discomfort, visual disturbances, and tear film instability. OSDs include dry eye disease (DED), blepharitis, meibomian gland dysfunction, keratitis, conjunctivitis, and related disorders. These diseases represent a leading cause of ocular morbidity and are often accompanied by chronic inflammation, irritation, redness, and pain. Royal Jelly (RJ), a substance produced by worker bees, has been widely studied in ophthalmology for its therapeutic properties, including its ability to restore tear secretion, treat glaucoma and DED, and inhibit the production of reactive oxygen species (ROS). RJ is rich in proteins, fatty acids, and phenolic compounds, which contribute to its anti-inflammatory, antioxidant, antibacterial, vasodilatory, antitumor, and cholesterol-lowering properties. This review examines the pharmacological benefits of RJ, strategies to optimize its formulation, and methods for developing eye drop formulations—such as microemulsions and eye gels—for the treatment of OSDs. The literature supports RJ as a complementary therapy for OSDs due to its reported anti-inflammatory, antioxidant, and antimicrobial properties. Although preliminary studies are promising, more extensive clinical trials are required to establish standardized treatment protocols and confirm the efficacy and safety of RJ. The therapeutic potential of RJ components lies in their immunomodulatory properties, making them a compelling option for the treatment of OSDs. Further research is necessary to clarify their role in ocular regenerative medicine and expand their applications in clinical practice.

##  INTRODUCTION

The growing interest in Royal Jelly (RJ) stems from its remarkable effects on human health, which has led to increased focus on the identification and analysis of its active components. A milky and viscous secretion produced by the mandibular and hypopharyngeal glands of young worker bees, RJ serves as food for larvae.^[1,2]^ During the larval period, the queen bee is fed with RJ, while this time is only 3 days for nurse bees.^[3]^

Several studies have explored the chemical composition and biological activities of RJ. Due to its unique biological properties, RJ has substantial clinical and commercial appeal and is currently used in the pharmaceutical, food, cosmetic, and manufacturing sectors.^[4,5]^

The composition of RJ is complex as it contains amino acids, organic acids, proteins, sugars, minerals, steroids, esters, phenols, trace elements, and other components.^[6]^ The main components of RJ include water (50–70%), lipids (3–8%), mineral salts (1.5%), proteins (9–18%), carbohydrates (7–18%), and small amounts of vitamins and polyphenols.^[2]^

Notably, > 80% of RJ proteins are soluble proteins such as major RJ protein 1 (MRJP1) to major RJ protein 8 (MRJP8), which play a pivotal role in specific physiological functions of RJ and have been fully characterized.^[7,8,9,10]^

In crude RJ protein, major RJ proteins (MRJPs) stimulate cell proliferation and inhibit the proliferation of bisphenol A-induced human breast cancer cell lines.^[11,12]^ Additionally, major RJ protein 3 (MRJP3) modulates immune responses by inhibiting the production of IL-4, IL-2, and IFN-c in T cells.^[13,14]^ These studies have shown that MRJP3 can have significant immunoregulatory effects, which could be important for developing new antiallergic peptides.^[5]^

It has recently been discovered that certain proteins and peptides exhibit antioxidative activity. Analysis of antioxidant peptides isolated from RJ hydrolysate showed potent hydroxyl radical scavenging activity.^[15]^ Three dipeptides (Lys-Tyr, Arg-Tyr, and Tyr-Tyr) from RJ hydrolysis showed strong hydrogen peroxide and hydroxyl radical scavenging activity.^[16,17]^ These dipeptides contain Tyr residues with phenolic hydroxyl groups at their C-termini, which are capable of scavenging free radicals by donating a hydrogen atom from their OH group.^[18,19]^ Additionally, RJ uniquely contains 10-HAD (10 carbon atoms), a fatty acid possessing various pharmacological effects.^[20]^ It has recently been discovered that RJ is also distinguished by a set of C8, C10, and C12 hydroxy fatty acids, and since RJ has a strong antibiotic effect, these fatty acids may contribute to some of the antibacterial peroxide activity of RJ.

**Figure 1:**
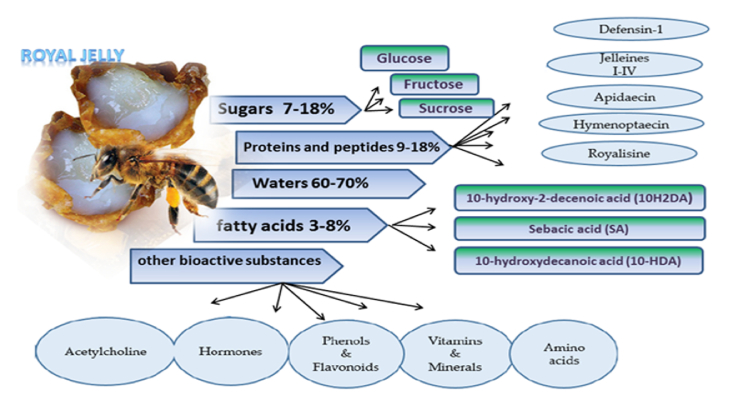
A comprehensive overview of the biological composition and bioactive components of RJ. The schematic illustrates the primary components of RJ and their diverse functional roles. The components include water content (60–70%), sugars (7–18%), proteins and peptides (9–18%), fatty acids (3–8%), and other bioactive compounds. Specific sugars include glucose, fructose, and sucrose; proteins and peptides include defensin-1, jelleines I–IV, apidaecin, hymenoptaecin, and royalisine; fatty acids include 10-hydroxy-2-decenoic acid (10H2DA), sebacic acid, and 10-hydroxydecanoic acid (10-HDA); and other bioactive substances include acetylcholine, hormones, phenols and flavonoids, vitamins and minerals, and amino acids. These components contribute to the antioxidant, anti-inflammatory, and antimicrobial properties of RJ.

Trace elements play a crucial role in the biomedical activities associated with RJ, as these elements have many known and unknown biological functions. Previous studies have explored the concentration of 28 trace elements and minerals in RJ samples.^[21,22,23,24]^ Ramadan and Al-Ghamdi revealed that RJ exhibits homeostatic regulation, similar to lactation at the insect level, and with similarities to the homeostatic mechanisms observed in mammalian and human breast milk.^[5]^ Figure 1 illustrates the RJ composition and the main functional activities of its compounds.

To identify and evaluate the scientific literature on RJ, we assessed the credibility of selected sources based on journal quality, research methodology, and sample size. These factors form the basis for claims regarding RJ composition and biological effects. We focused on specific active components, such as MRJP proteins, their functions, antioxidant properties, and the role of trace elements in RJ's health benefits. Additionally, we ensured the reliability of findings by noting consistency across multiple studies regarding the physiological roles and therapeutic potential of RJ.

### Formulation of Ocular Gels with RJ 

Freshly harvested RJ should be stored at low temperatures, but it is recommended to integrate it into pharmaceutical formulations to increase stability. One of the most biologically active compounds in RJ is trans-10-hydroxy-2-decenoic acid (10-HDA), a component found exclusively in RJ.^[25]^ The 10-HDA has various biological activities, including anti-inflammatory, antimicrobial, and antioxidant properties.^[26,27]^ Data related to the ocular use of RJ indicate that RJ and 10-HDA supplements have a positive effect in treating dry eye syndrome and corneal alkali burns. RJ and 10-HDA have different biological activities and are stored in a freezer (–18ºC).

*In situ* gel formation is one of the most suitable methods to stabilize and preserve these biological activities.^[28,29]^ These gels are ophthalmic drug delivery systems that improve ocular bioavailability and ensure longer pre-corneal residence time, compared to conventional topical drops.^[30]^ These *in situ* gels form only when applied to the conjunctival sac, creating a viscoelastic gel.^[31]^ Among the materials used for such formulations, poloxamers—known for their transparency, safety, and good aqueous solubility—are commonly used in ophthalmic drug formulations due to their favorable properties for semisolid preparations.^[32,33]^ On the other hand, RJ has antimicrobial activity as it suppresses the growth of *Escherichia coli*, *Pseudomonas aeruginosa*, and *Candida albicans*. RJ has a significantly lower impact on the *Staphylococcus aureus* strain. Besides, RJ has exhibited the least antibacterial activity against *Bacillus cereus*.^[29]^

The results of many studies indicate that RJ is a promising candidate for eye drops.^[29]^ *In situ* gel formulations prepared with RJ are suitable for ophthalmic applications, as they exhibit longer ocular residence time and bioavailability compared to conventional eye drops.^[34]^ When applied to the surface of the eye, these solutions transform into a gel, creating a layer on the surface that resembles a temporary lens.^[35]^ Previous studies have shown that *in-situ* gel formulations do not cause cell death when used in small amounts for 24 hours. These formulations can be safely utilized as nonirritant and nontoxic alternatives, confirming their safety for ocular applications.^[36,37]^ In one study, evaluating the antioxidant and antimicrobial activities showed that Lithuanian RJ exhibits strong antimicrobial activity. Additionally, the *in situ* gels containing RJ were nontoxic and had suitable physicochemical properties for ophthalmic preparations.^[29]^

### Therapeutic Potential of RJ in Ocular Surface Diseases (OSDs)

#### Dry eye disease (DED)

DED is characterized by an unstable tear film that leads to ocular surface damage and visual impairment.^[38]^ Due to the use of digital devices, the prevalence of DED has increased.^[39]^ Treatment with artificial tears can provide temporary relief, but it is not a permanent solution as it does not address the underlying causes. Previous research has shown that RJ can enhance tear production and reduce adenosine triphosphate (ATP) content and mitochondrial levels in the lacrimal gland (LG), demonstrating its potential as a proactive approach to addressing DED.^[40]^ RJ contains acetylcholine (ACh) and fatty acids, which are associated with various health benefits, including anti-inflammatory and antimicrobial effects.^[40]^ Furthermore, RJ contains ACh at a concentration of 1 mg/g and has the potential to restore tear secretion.^[41]^ Some of these fatty acids have been associated with various pharmacological activities such as anti-inflammatory, anticancer, and antimicrobial properties.^[42,43]^ Although RJ is composed of C8, C10, and C12 fatty acids,^[44]^ 10-hydroxydecanoic acid (10HDAA) and (E)-10-Hydroxy-2-decenoic acid (10H2DA) are known to make up 60–80% of its lipid content.^[45]^ These fatty acids activate the TRPA1 channels in HEK293 cells, enhance filaggrin production in a 3D human epidermis model,^[46]^ induce estrogen receptor in MCF-7 cells,^[47]^ and express GLUT4 in skeletal muscle.^[46]^ However, it has been shown that orally administered RJ has ACh-like effects, such as improving Alzheimer's disease.^[48]^ Another report showed that RJ has the ability to secrete tears through muscarinic acetylcholine receptor (mAChR) signaling.^[41]^ These reports indicate that after oral administration of RJ, the ACh in RJ retains its activity. This is probably because RJ fatty acids prevent ACh degradation by AChE and efficiently transport ACh to targeted sites.^[49]^ On the other hand, a previous study confirmed the interaction of decanoic acid with mAChR agonists. Therefore, 10HDAA, 8HOA, and 3,10DDA could also interact with mAChR.^[49]^ Also, 3,10DDA, 8HOA, and 10HDAA could play the role of mAChR modulators for binding allosteric sites and/or stabilizing lipid membranes.^[49]^

#### Corneal ulcers 

A corneal ulcer refers to an inflammatory and, in more severe cases, infectious disease of the cornea. This involves disruption of the corneal epithelial layer, often involving the corneal stroma. The disease plays a significant role in monocular blindness in many developing countries throughout Africa, Asia, and the Middle East, especially after untreated cataracts.^[50,51]^ This vision-threatening condition affects men and women and different age groups globally. Risk factors for corneal ulcers include ocular surface disease (OSD), trauma, immunosuppression, contact lens use, recent corneal surgery, and long-term use of topical medications.^[52]^

Various types of honey and RJ have been used experimentally in ophthalmology to treat OSDs, such as corneal burns, in animal models.^[53,54,55,56]^ Chestnut honey (CH), native to Turkey, is commonly used in complementary medicine for various conditions. The results indicate that RJ shows potential for treating corneal ulcers and some other ophthalmic diseases. RJ has exhibited anti-inflammatory characteristics in numerous conditions associated with abnormal inflammatory processes.^[57]^ For instance, Chinese researchers studied the anti-inflammatory effects of RJ on the BV-2 murine microglial cell line exposed to lipopolysaccharides (LPS), which are recognized to induce inflammation. It was found that RJ exerted a protective influence on the cells, mitigating the inflammatory response. The potential mechanisms could be linked to the inhibition of pro-inflammatory cytokine synthesis. RJ significantly inhibited the expression levels of pro-inflammatory protein COX-2 and also hindered nuclear factor kappa B pathways, thereby reducing inflammation.^[58]^ The application of RJ in the treatment of corneal ulcer has shown promising results in some studies. However, further research, particularly involving human participants, is needed to fully establish the effectiveness and safety of RJ in the treatment of these eye diseases.

### Oral Administration of RJ and Tear Secretion

The ocular surface is covered by a thin layer of an aqueous tear film that maintains homeostasis.^[59,60]^ This film smooths the cornea and acts as the first defense system against drying, environmental microbes, and foreign bodies.^[61,62]^ Extensive use of digital devices can alter the blinking dynamics and aggravate dry eye symptoms. Aging also decreases LG function and plays a significant role in inducing dry eye syndrome.^[63,64]^ Finding strategies to maintain healthy LG function may have significant clinical implications. The main products of beekeeping, such as honey, pollen, RJ, and propolis, are deeply rooted in various cultures and are widely used for their health benefits.^[40]^ In traditional medicine, honey eye drops have been used for corneal ulcers, and propolis has been applied to reverse retinal damage.^[65,66]^

Recent studies about the effects of bee products on tear secretion capacity in dry eye syndrome have used a blink-inhibited dry eye model. The studies demonstrate that RJ evokes protein secretion from the LG, a critical function for tear secretion. Furthermore, in a dry eye model, RJ effectively restored decreased tear secretion.^[67,68]^ Additionally, dry eye, characterized by clinical manifestations, such as changes in the ocular surface, particularly reflects corneal epithelial damage that can be reduced by RJ administration. These results indicate the direct effect of RJ on LG function, making it a potent nutritional treatment for the prevention of dry eye.^[40]^

Previous research has shown that LG produces tear fluid by combining transepithelial water flow from an osmotic gradient with the release of proteins from intracellular vesicles.^[69,70]^ To facilitate the osmotic gradient, numerous ion transporters and water channels are activated. The process of secretory vesicle-mediated exocytosis from LG cells requires an energy source of ATP to create protein-rich aqueous fluid.^[71,72,73]^ One study showed that stressful conditions in a dry eye model resulted in reduced tear secretion capacity, decreased ATP levels, impaired secretory system function, decreased mitochondrial content in the LG, and impaired lacrimal function.^[40,74,75]^ This suggests that the reduction in LG energy status under dry eye conditions significantly impairs tear secretion capacity. Conversely, increased cytosolic free calcium levels activate adenosine monophosphate-activated protein kinase (AMPK) in response to Ca²⁺.^[76,77]^ These studies show that oral administration of RJ increases tear secretion capacity and elevates mitochondrial levels, ATP, and AMPK phosphorylation in the LG. In LG acinar cells, Ca²⁺ mobilization stimulates tear fluid secretion.^[40,78]^ RJ contains substances that help maintain ATP levels by increasing cytosolic Ca²⁺. By activating muscarinic receptors, ACh increases intracellular Ca2+ levels.^[79]^ Additionally, by activating nonselective Ca²⁺-permeable cation channels, 10-hydroxy-2-desnoic acid induces extracellular Ca²⁺ entry. However, 10-HDE does not stimulate Ca²⁺ mobilization and acts as a muscarinic receptor inhibitor. These findings suggest that through the muscarinic signal transduction pathway, RJ potentially mobilizes Ca²⁺, maintains energy status, and restores tear secretion capacity. This indicates that oral RJ restores tear secretion capacity in dry eye by stimulating the calcium signaling pathway and affecting the energy status of the LG.^[40]^

### Biopharmaceutical Evaluation of Ophthalmic Microemulsions Containing RJ

One way to enhance stability in ocular drug delivery is by incorporating RJ into microemulsions. Conventional liquid ophthalmic formulations are characterized by poor bioavailability due to rapid drug clearance from the pre-corneal area caused by tear secretion and nasolacrimal drainage.^[80]^ Microemulsions, consisting of co-surfactants, oils, surfactants, and water, provide a promising solution by ensuring drug stability, controlled release, and improved solubility.^[81,82]^ However, selecting nonirritating components remains a key challenge.^[82]^

RJ-based ocular microemulsions have been studied for their quality and biological activity.^[83]^ The most significant component in RJ, 10-HDA, with concentrations ranging from 1% to 3%, contributes to its antioxidant properties.^[83,84]^ RJ also contains amino acids such as arginine, histidine, and lysine, which exhibit strong antioxidant activity.^[85]^ These properties suggest that RJ could serve as a natural source of amino acids for eye drops, aiding in OSD treatment.^[86]^ Additionally, microemulsions containing RJ have a pH ranging from 5.68 to 6.89, which should ideally be closer to the physiological pH of tears (7.2).^[81]^ Liquid formulations like microemulsions enhance drug bioavailability due to their spreading and wetting properties. They can solubilize both hydrophilic and lipophilic drug molecules, making them suitable for sustained ocular drug delivery.^[87]^ Isopropyl myristate is often included in the oil phase due to its stability and eye tolerance.^[88]^ Studies show that RJ and 10-HDA reduce reactive oxygen species (ROS) without inducing cell toxicity, making them promising candidates for antioxidant eye drops.^[89]^ By optimizing formulation components, RJ-based microemulsions offer a stable, effective, and nontoxic approach for treating ocular conditions while improving drug retention and therapeutic efficacy.^[90,91]^

### Preclinical and Clinical Studies of RJ in OSDs

Although data on the ocular use of RJ are limited, studies support its potential benefits for dry eye syndrome and corneal healing.^[92,93]^ In a recent study, Perminaite et al developed *in situ* ocular gels containing Lithuanian RJ to improve corneal retention and bioavailability. The gels were evaluated for pH, rheology, refractive index, and 10-HDA release, and the results showed acceptable pH and refractive index values. The gelation temperature ranged from 25 to 32ºC, depending on poloxamer concentration. Additionally, 10-HDA release was more sustained compared to the RJ suspension. The formulations were nonirritant and did not induce cell death, suggesting their potential for stable and safe ocular drug delivery.^[29]^

Recent studies highlight the detrimental effects of oxidative stress on the ocular surface and its crucial role in DED.^[94]^ Biomarkers of oxidative damage, such as lipid peroxidase and ROS, have been identified in the tears and conjunctival tissues of patients with DED.^[95]^ This oxidative imbalance, worsened by UV radiation and environmental pollutants, disrupts ocular homeostasis. RJ has shown promising antioxidant and anti-inflammatory properties, which may help counteract oxidative stress. Studies indicate that oral RJ supplementation enhances tear secretion in dry eye models induced by reduced blinking.^[40]^ Additionally, RJ intake was shown to boost ATP production and improve mitochondrial function by regulating the calcium signaling pathway. This suggests that RJ may support LG activity and help restore tear production, offering potential therapeutic benefits for DED.^[40]^ The study by Atalay et al investigated the effect of endemic CH and RJ on corneal healing after an alkaline burn in Wistar rats. The rats were treated with RJ, CH, a combination of RJ and CH, or Na-Hyaluronate (Na-HA) eye drops. Although no significant differences in healing scores were found on the 1 ${}^{st}$ , 7 ${}^{\text{th}}$ , and 14 ${}^{\text{th}}$ days, the RJ–CH and CH groups showed improved healing outcomes in repeated measures. Additionally, higher levels of α 4 β 1 integrin were observed in the treatment groups. The study concluded that RJ–CH eye drops promoted corneal healing, as indicated by healing scores and integrin staining.^[96]^ It was found that tissue inhibitors of matrix metalloproteinases (MMPs) were as effective as synthetic MMP inhibitors in improving corneal ulcers in a rabbit model of corneal alkali burn. Majtan et al investigated the effects of MRJP1—a key active component of RJ and acacia honey—on MMP mRNA expression in human keratinocytes. They observed increased MMP mRNA expression in the acacia honey group, but no such induction in the MRJP1 group.^[97]^ RJ demonstrated beneficial effects in a rat model of aqueous tear deficiency (ATD), which involved exposure to low room temperature, low humidity, constant airflow, and placement on a swing to reduce blinking. Rats received daily oral administration of 1 mL of distilled water (vehicle) or RJ, honey, pollen, larva, or propolis for 11 days. RJ treatment resulted in the highest increase in lacrimal protein secretion ( Δ RJ: +175% vs. Δ vehicle: +60%; *P* < 0.001) and tear secretion (RJ: +1.2 × vs. vehicle: +0.5 × ; *P* < 0.001) compared to all other compounds.^[40]^

A randomized controlled trial assessed the effects of RJ on 43 individuals with a deficiency. Participants received either 400 mg RJ tablets (six times daily) or a placebo for 8 weeks. The RJ group showed greater improvements in tear stability (TBUT: 4.5 ± 3.2 s to 6.2 ± 2.9 s) and tear production (Schirmer test: 13.6 ± 10.6 mm to 19.5 ± 11.7 mm), though between-group differences were not statistically significant. No adverse effects were reported. These findings suggest RJ may support tear film stability and secretion, warranting further research into its potential as a therapeutic option for ATD.^[28]^ These findings suggest that RJ may enhance LG activity, potentially by increasing its energy content.

In summary, while research on the ocular use of RJ remains limited, existing studies provide compelling evidence for its therapeutic potential in treating DED and promoting corneal healing. The antioxidant and anti-inflammatory properties of RJ, along with its ability to enhance tear secretion and support LG function, suggest significant benefits in managing ocular surface disorders. Moreover, recent preclinical and clinical studies highlight RJ's promising effects in improving tear production, tear film stability, and corneal healing, with no severe adverse effects reported. These findings indicate that RJ may be a valuable adjunct in the treatment of DED and other ocular conditions. However, further research is needed to fully establish its clinical efficacy and optimal application.

### The Influence of RJ on Stem Cells 

**Figure 2:**
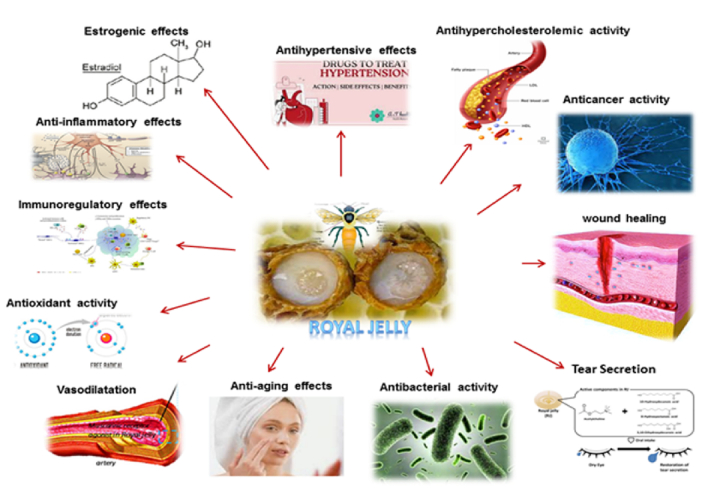
Therapeutic and biological properties of RJ: antioxidant, anti-inflammatory, immunoregulatory, antibacterial, anticancer, estrogenic effects, wound healing, tear secretion enhancement, anti-aging, vasodilation, cholesterol-lowering, and antihypertensive effects.

#### (i) In vitro studies 

RJ contains exosome-like vesicles that act on signaling pathways, contributing to its antibacterial and pro-regenerative effects. These vesicles exhibit bacteriostatic, bactericidal, and biofilm-inhibiting properties and are internalized by mesenchymal stem cells (MSCs), influencing their migration potential.^[98]^ In wound-healing assays, RJ vesicles enhance MSC migration, differentiation, and secretome modulation, while reducing inflammation in macrophages via MAPK pathway inhibition.^[99,100]^ The impact of RJ on stem cells, both normal and cancerous, highlights its regenerative and immunomodulatory potential. RJ supports innovative treatment strategies for degenerative diseases, such as sarcopenia, by enhancing muscle satellite cell function. Additionally, RJ shows promise in bone engineering and regenerative medicine through its effects on MSCs. RJ supplementation in Wharton's jelly MSC increases mitochondrial numbers, reduces senescence, and enhances osteogenic differentiation.^[101,102]^ Neural stem cell studies show RJ extract promotes differentiation into neurons, astrocytes, and oligodendrocytes, with components like 10-hydroxy-trans-2-decenoic acid (HDEA) facilitating neurogenesis. AMP N1-oxide, another RJ component, stimulates neurite growth in cultured PC12 cells and supports fibroblast growth factor activity, enhancing neural stem cell proliferation.^[15]^ RJ influences embryonic stem cells through royalactin, which sustains pluripotency by activating ground-state pluripotency-like networks.^[103]^ Studies on goat oocyte *in vitro* maturation show RJ improves embryo production, enhances maturation rates, and reduces apoptosis-related gene expression.^[104,105]^ Studies on bone marrow stem cells indicate RJ's potential as a biological response modifier with myeloprotective and antitumor effects.^[106]^ RJ also positively differentiates embryonic carcinoma P19 cells into neurons.^[107]^ RJ's components, such as AMP N1-oxide, promote astrocyte production in neural progenitor cells, enhance progenitor cell proliferation, and encourage the expression of mature nerve cell proteins.^[108]^ Figure 2 depicts the therapeutic and biological characteristics of RJ.

#### (ii) In vivo and clinical studies 

Recent studies have highlighted the effects of RJ on stem cells, particularly focusing on its role in maintaining the pluripotency of embryonic stem cells in animal models. *In vivo* experiments underscore RJ's regenerative and therapeutic effects. In a mouse model, RJ exhibited antibacterial properties and accelerated wound healing.^[99]^ Integrated into type I collagen gels, RJ vesicles enhanced fibroblast uptake, migration, and contraction, facilitating the wound-healing process.^[109]^ In aged mice, RJ treatment attenuated muscle weight and grip strength decline, improved regenerative capacity, and increased serum insulin-like growth factor-1 levels.^[40]^ RJ's positive effects on muscle regeneration include reducing inflammation and oxidative stress, regulating metabolism, enhancing stem cell responsiveness, and promoting nerve regeneration.^[110]^ Animal studies on mouse models of stroke indicate that the combined administration of mMSC + RJ improves motor function and reduces inflammation-associated risks, offering a potential strategy to mitigate long-term complications associated with stem cell therapy.^[111]^{Tan,2022 #367} Neural studies revealed RJ's ability to regenerate TMT-injured hippocampal tissue, improving neuronal function and cognition.^[57]^ RJ supplementation in cancer stem cell models demonstrated anti-tumorigenic effects, particularly through RJ-protein fraction 50 (PF50) and major RJ protein 2 (MRJP2), both of which exhibited anti-leukemic activity.^[25]^ RJ also prevented myelosuppression and supported splenic hematopoiesis in Ehrlich ascites tumor-bearing mice.^[26]^

**Table 1:** Therapeutic applications of different active pharmaceutical ingredients of royal jelly

**Disease**	**Cell/System**	**Active pharmaceutical ingredient**	**Advantage**	**Outcome**	**Reference**
Dry eye	Corneal cell	10 HDA - RJ	Nontoxic	Reduction of intracellular ROS; no induction of cell death after exposure to dryness	^[40]^
Dry eye	Human	RJ	Safe and effective in increasing tear volume	Preservation of tear secretion; activation of LG secretory function	^[28]^
Dry eye	Rat	Honey, propolis, RJ, pollen	Restoration of tear secretion capacity; reduction in LGs ATP content	Preventative intervention for dry eye; managing tear secretion capacity in the LGs	^[40]^
Glaucoma	Retinal ganglion cells	RJ antioxidants	Neuroprotective	Prevention of retinal ganglion cell apoptosis and slowing progression of neurodegeneration	^[112]^
Corneal alkali burns	Corneal epithelial cells	Corneal epithelial cells, RJ, especially 10-hydroxy-2-decenoic acid (10-HDA)	Antioxidant, anti-inflammatory, and antibacterial effects, *in situ* gel formulation for longer eye surface retention, sustained release of 10-HDA, nonirritant, and safe in cell tests	Enhanced stability and bioavailability, nonirritant formulation, safe for corneal cells, potential for treating corneal alkali burns	^[113]^
RJ, royal jelly; 10-HAD, 10-hydroxy-2-decenoic acid; ROS, reactive oxygen species; LG, lacrimal gland; ATP, adenosine triphosphate					

While much of the research surrounding RJ has been conducted using *in vitro* and *in vivo* models, preliminary human clinical trials are beginning to explore its effects on stem cells. A randomized, double-blind, placebo-controlled trial involving healthy participants revealed that RJ administration may influence peripheral hematopoietic stem cell (HSC) counts and stem cell functions, prompting further research into its health-promoting benefits.^[44]^ These studies demonstrate the diverse potential of RJ in addressing various health conditions. However, further research is needed to establish optimal dosing, duration, and specific applications for different patient populations.

Table 1 outlines some of the active pharmaceutical ingredients of RJ, their application in the treatment of some diseases, and their remarkable results.

##  Summary

RJ shows great promise as a natural therapeutic option for managing OSDs. Its antioxidant, anti-inflammatory, and immune-modulating properties protect the ocular surface, promote healing, and enhance overall eye health. RJ is particularly valuable as an adjunctive therapy for conditions such as dry eye syndrome, corneal ulcer, conjunctivitis, and keratitis. Its effects on stem cells highlight its potential in ocular regenerative medicine, with RJ vesicles enhancing migration, differentiation, and regenerative capacity in MSCs, thereby supporting wound healing and tissue repair. The development of innovative formulations, such as microemulsion and eye gel, optimizes RJ delivery to the ocular surface and thus improves its therapeutic efficacy. While preliminary findings are promising, further research, including well-designed clinical trials, is essential to confirm RJ's efficacy and safety in OSD treatment. Additionally, standardized formulations and dosing protocols are needed to ensure consistent outcomes. The unique bioactive components of RJ offer significant potential for advancing treatment strategies and improving ocular health in the future.

##  Financial Support and Sponsorship

None.

##  Conflicts of Interest

None.
